# Effect of hypothyroidism on contractile performance of isolated end-stage failing human myocardium

**DOI:** 10.1371/journal.pone.0265731

**Published:** 2022-04-11

**Authors:** Nancy S. Saad, Mohammed A. Mashali, Mohammad T. Elnakish, Austin Hare, Courtney M. Campbell, Salome A. Kiduko, Kyra K. Peczkowski, Amanda W. Huang, Farbod Fazlollahi, Gina S. Torres Matias, Amany A. E. Ahmed, Bryan A. Whitson, Nahush A. Mokadam, Paul M. L. Janssen

**Affiliations:** 1 Department of Physiology and Cell Biology, College of Medicine, The Ohio State University, Columbus, OH, United States of America; 2 Davis Heart and Lung Research Institute, The Ohio State University, Columbus, OH, United States of America; 3 Faculty of Pharmacy, Department of Pharmacology and Toxicology, Helwan University, Cairo, Egypt; 4 Faculty of Veterinary Medicine, Department of Surgery, Damanhour University, Damanhour, Egypt; 5 Division of Cardiovascular Medicine, Department of Internal Medicine, The Ohio State University, Columbus, OH, United States of America; 6 Division of Cardiac Surgery, College of Medicine, The Ohio State University, Columbus, OH, United States of America; York University, CANADA

## Abstract

The relationship between hypothyroidism and the occurrence and progression of heart failure (HF) has had increased interest over the past years. The low T3 syndrome, a reduced T3 in the presence of normal thyroid stimulating hormone (TSH), and free T4 concentration, is a strong predictor of all-cause mortality in HF patients. Still, the impact of hypothyroidism on the contractile properties of failing human myocardium is unknown. Our study aimed to investigate that impact using *ex-vivo* assessment of force and kinetics of contraction/relaxation in left ventricular intact human myocardial muscle preparations. Trabeculae were dissected from non-failing (NF; *n* = 9), failing with no hypothyroidism (FNH; *n* = 9), and failing with hypothyroidism (FH; *n* = 9) hearts. Isolated muscle preparations were transferred into a custom-made setup where baseline conditions as well as the three main physiological modulators that regulate the contractile strength, length-dependent and frequency-dependent activation, as well as β-adrenergic stimulation, were assessed under near-physiological conditions. Hypothyroidism did not show any additional significant impact on the contractile properties different from the recognized alterations usually detected in such parameters in any end-stage failing heart without thyroid dysfunction. Clinical information for FH patients in our study revealed they were all receiving levothyroxine. Absence of any difference between failing hearts with or without hypothyroidism, may possibly be due to the profound effects of the advanced stage of heart failure that concealed any changes between the groups. Still, we cannot exclude the possibility of differences that may have been present at earlier stages. The effects of THs supplementation such as levothyroxine on contractile force and kinetic parameters of failing human myocardium require further investigation to explore its full potential in improving cardiovascular performance and cardiovascular outcomes of HF associated with hypothyroidism.

## Introduction

According to the American College of Cardiology, heart failure (HF) is a complex clinical syndrome that impairs the ability of the ventricle to fill with or eject blood [1]. HF represents a common final condition of several cardiac diseases [2]. The prevalence of HF continues to rise over time with the aging population. An estimated 6.2 million American adults ≥ 20 years of age (2.2%) had HF between 2013 and 2016 compared with an estimated 5.7 million between 2009 and 2012 [3]. Despite progress in treating HF over the past 15 years, the estimated increases in the morbidity and mortality rates is likely partially due to our lack of adequate understanding of the pathophysiological mechanisms of this syndrome [4].

Thyroid hormones (THs) play essential roles in cardiovascular homeostasis [5]. THs affect every structure of the heart and its specialized conducting system. T3 is the biological active thyroid hormone; in peripheral tissues T3 is mostly generated by 5′-monodeiodination of thyroxine (T4) [6]. Multiple experimental and clinical studies have focused on the relationship between thyroid hormone and the cardiovascular system [7–12]. The physiological effects of thyroid hormone are mostly mediated by its genomic nuclear effects through binding to thyroid hormone nuclear receptors (TRs). The occupancy of these receptors by T3 modify the rate of transcription of specific target genes [7,8] and in the absence of T3 they repress transcription [13]. Some of the important cardiac structural and functional proteins that are transcriptionally regulated by T3 are alpha myosin heavy chain (αMHC), voltage-gated K^+^ channels, β1 adrenergic receptors, malic enzyme and atrial and brain natriuretic hormone [7,8] while βMHC, phospholamban, the Na^+^/Ca^2+^ exchanger, and adenylyl cyclase type V and VI are negatively regulated genes that are repressed in the presence of T3 [7,8]. Thyroid hormone also upregulates sarcoplasmic reticulum calcium-activated ATPase 2 (SERCA2) and Na^+^/K^+^ ATPase and downregulates phospholamban [14]. These changes enhance contractile function and diastolic relaxation in the heart [14]. Moreover, T3 influences the sensitivity of the sympathetic system by increasing the number of myocardial adrenergic membrane receptors. T3 also leads to hemodynamic alterations in the periphery by decreasing systemic vascular resistance through relaxation of vascular smooth muscle. Renal perfusion as a consequence decreases and leads to activation of renin-angiotensin aldosterone system that leads to increased cardiac filing and modification of cardiac contractility [15,16]. Thyroid hormone enhances the release of vasodilatory mediators as a result of increasing metabolic and oxygen consumption [15]. Increasing resting heart rate and left ventricular contractility mediated by T3 are partially depending on its non-genomic mechanisms [17]. These effects of TH start very quickly and do not require the binding to nuclear receptors [18], otherwise they involve the transport of ions (calcium, sodium and potassium) across the plasma membrane, mitochondrial function, glucose and amino acid transport, and a variety of intracellular signaling pathways [8,18].

Disease of the thyroid gland often produces cardiac problems; it can worsen cardiac disease symptoms or can accelerate the underlying cardiac problem in a heart disease person as well as producing cardiac disease in people with healthy hearts. Untreated subclinical hyperthyroidism and hypothyroidism were found to be associated with the development of HF in diseased or healthy heart patients [19,20]. In the US general population, hypothyroidism is more prevalent than hyperthyroidism: 4.6% (0.3% clinical and 4.3% subclinical) compared to 1.3% (0.5% clinical and 0.7% subclinical), respectively, suggesting that low thyroid hormone conditions could be worth more concern. Nearly 13 million Americans are estimated to have undiagnosed hypothyroidism [21].

Decreased THs are considered to play a role in the progression of the HF since cardiac contractility, energy metabolism, and myocyte shape, as well as angiogenesis and fibrosis are all regulated by T3 [22–24]. Patients with hypothyroidism usually exhibit impaired ventricular relaxation related to reductions in the activity of the Ca^2+^-ATPase within the sarcoplasmic reticulum, increased expression of phospholamban, and associated reductions in intracellular calcium reuptake during diastole [8,25,26]. Moreover, both systolic and diastolic functions are impaired during effort, leading to decreased exercise tolerance for the slowed myocardial relaxation and the impaired left ventricular filling and vasodilatation during effort [9,27]. These hemodynamic abnormalities likely contribute to the heightened risk of incident HF associated with subclinical hypothyroidism as shown in an individual patient meta-analysis of the Thyroid Studies Collaboration [28]. Alterations in gene expression developed in the failing heart were found to be similar to the alterations induced in experimental hypothyroidism, suggesting that TH dysfunction has an important role in the progression of HF [29]. The low T3 syndrome, a reduced T3 in the presence of normal thyroid stimulating hormone (TSH), and free T4 concentration, is a strong predictor of all-cause mortality in HF patients [30].

In this study, we aimed to investigate the impact of hypothyroidism on force and kinetics of contraction/relaxation of failing myocardium obtained from patients with combined HF and hypothyroidism. This study could potentially help understand the influence of hypothyroidism on such hearts from the contractile point of view, which may help future studies find an appropriate targeted treatment that can improve cardiovascular performance and cardiovascular outcomes of such cases.

## Materials and methods

### Human tissue procurement

All human tissues studies were conducted with approval from the Institutional Review Board (IRB) of The Ohio State University and conform to the Declaration of Helsinki. In collaboration with Lifeline of Ohio Organ Procurement Program, non-transplantable donor hearts were acquired in the operating room. All end-stage failing hearts were acquired from patients undergoing cardiac transplantation at The Ohio State University Wexner Medical Center. Informed consents were obtained from cardiac transplant patients. Immediately after removal from the patients/donors, hearts were flushed with cardioplegic solution as described previously [31,32]. Over the past 11 years, a total of 214 hearts were procured. Of these, 55% (*n* = 119) were from patients who had end-stage HF, while the remaining (*n* = 95) were from donors with no apparent history of HF. Retrospective analysis of our data showed that 20% of HF patients (*n* = 24) had a history of hypothyroidism. Of these, contractility experiments were successfully performed, and data were collected from 9 procured hearts. Hence, contractile force and kinetics were comparatively assessed in myocardium dissected from: HF patients with a history of hypothyroidism (FH; *n* = 9), HF patients with no history of hypothyroidism (FNH; *n* = 9), and non-failing donors (NF; *n* = 9). Donor/patient demographics and hearts’ dimensions and ventricular wall thickness are provided in Tables 1 and 2.

**Table 1:** Donor demographic and characteristics of procured human hearts (NF; *n* = 9).

ID #	Sex	Age	Race	BMI	HW (g)	Overall dimensions (cm) Length, Width, Depth			LV Wall (cm)	RV Wall (cm)	Septum (cm)
NF1	M	58	C	32.1	512	13.5	10	9.5	1.7	0.9	1.9
NF2	F	62	C	35.5	478	14.5	10.5	9.5	1.6	0.7	1.7
NF3	M	23	C	33.9	462	9.5	14	9.5	1.8	0.7	1.6
NF4	F	38	C	31	406	9.5	10	9.5	2.3	0.6	1.8
NF5	M	20	C	25.9	324	9	13.3	8.5	1.8	1	1.4
NF6	F	55	AA	24.5	350	8.5	11.5	8.5	1.5	0.4	1.8
NF7	F	51	C	20.6	507	13.5	10	11.5	2	0.9	1.4
NF8	M	67	C	29.2	527	15.3	11.8	11	2.2	0.8	2.9
NF9	F	43	AA	20.9	605	10.5	13.5	10	2	0.8	2

AA, African American; BMI, body mass index; C, Caucasian; F, female; g, gram; HW, heart weight; LV, left ventricle; M, male; NF, non-failing; RV, right ventricle.

**Table 2:** HF patient demographics and characteristics of procured human hearts (*n* = 18).

ID #	Failing category	Sex	Age	Race	BMI	HW (g)	Overall Dimensions (cm) Length, Width, Depth			LV Wall (cm)	RV Wall (cm)	Septum (cm)
**Failing human hearts with no hypothyroidism (FNH; *n* = 9)**												
FNH1	ICM	M	57	C	30	619	10.5	11	10.5	1.2	0.9	1.1
FNH2	NICM	F	32	C	26	435	14.4	10.9	8	1.1	0.6	0.9
FNH3	NICM	M	49	C	31.3	1153	21	16	15	1.6	1	1.6
fNH4	NICM	M	59	C	27	972	14.5	16	14	1.5	0.5	1.7
fNH5	NICM	M	56	C	25.5	474	11	12.5	9.5	1.1	0.6	0.8
fNH6	NICM	M	40	C/AA	25.6	747	12	15	11	1.4	0.9	1.3
fNH7	NICM	F	39	AA	33.9	365	10.7	13.5	10	1.3	0.8	1
fNH8	NICM	F	51	C	28.5	572	-	-	-	2.5	1	2.4
fNH9	NICM	M	64	C	23.3	564	11	14	9.5	2.2	1.2	1.5
**Failing human hearts with hypothyroidism (FH; *n* = 9)**												
FH1	ICM	M	64	C	27.4	-	-	-	-	-	-	-
Fh2	ICM	M	65	C	24.5	621	12.5	11	8.5	1.9	0.9	0.8
FH3	ICM	M	52	C	23	498	14	12	11	1.5	0.7	1.3
FH4	ICM	F	50	C	33.5	486	14	13	11	1.2	0.6	1.8
FH5	ICM	M	59	C	32.5	530	10.5	10.5	11	1.5	0.8	1.1
FH6	NICM	M	63	C	23.5	329	14	10	10	1.5	1	1.1
FH7	NICM	M	68	C	25.6	768	16	15	14	1.4	0.8	1.2
FH8	NICM	M	52	C	28.3	390	10.5	10	8.5	1	0.7	1.3
FH9	NICM	F	35	C	28.7	262	7.5	9	7	1.2	0.7	0.8

C/AA, Caucasian/African American; FH, failing with hypothyroidism; FNH, failing with no hypothyroidism; HF, heart failure; ICM, ischemic cardiomyopathy; NICM, non-ischemic cardiomyopathy; other abbreviations as in Table 1.

In a recent study from our group, studying the impact of etiology on force and kinetics of left ventricular end-stage failing human myocardium, failing myocardium showed a relatively slower kinetics when stimulation frequency increased, muscle length stretched, and isoproterenol concentration increased (compared to non-failing) with a markedly slowing down of relaxation kinetics in myocardium from failing hearts of NICM origin [33]. The etiology disproportion between the two failing groups in our study, didn’t impact the contractile force outcomes as well as the kinetics parameters.

### Trabecula isolation

For each heart, the left ventricle (LV) was rapidly transferred from the cardioplegic solution to a cold modified Krebs-Henseleit solution (K-H) containing 2,3-butanedione monoxime (BDM) [34], where uniform linear trabeculae were dissected by the aid of a stereo dissection microscope. Muscles were transferred into custom-made setups as previously described [35], and the perfusion solution was exchanged for a BDM-free K-H solution, circulating at 37°C. Stimulation was started at a baseline frequency of 0.5 Hz, and the CaCl_2_ concentration of the solution was gradually raised to 2 mM over ~15 minutes. Muscles were gradually stretched (over a few minutes) to an optimal length (L_100_ or L_opt_), where a small increase in length resulted in nearly equal increase in active developed tension and resting tension. This length roughly corresponds to sarcomere length of ~2.2 μm, which is near or at the *in-vivo* sarcomere length at end-diastole [31,32].

### Baseline trabeculae function

Baseline contractile force and kinetics of contraction and relaxation were assessed at a frequency of 1 Hz once muscles had stabilized in the set-up. This frequency is close to the physiological normal resting *in-vivo* heart rate of ~60–70 bpm. The dimensions of muscles were measured under the stereo microscope (at 40x magnification, resolution of ~10 μm), and all recorded forces were normalized to the cross-sectional area of the trabecula. Average dimensions (width × thickness × length) of LV trabeculae from NF, FNH, and FH were (0.35 ± 0.03 × 0.23 ± 0.02 × 3.74 ± 0.42 mm), (0.53 ± 0.1 × 0.36 ± 0.06 × 2.74 ± 0.38 mm), and (0.46 ± 0.06 × 0.31 ± 0.04 × 2.9 ± 0.4 mm) respectively. There were no significant differences between the dimensions of the muscles of the three studied groups.

### Assessment of contractile properties and kinetics of relaxation

The three main physiological modulators that regulate contractile strength: length-dependent activation, frequency-dependent activation, and β-adrenergic stimulation were assessed under near-physiological conditions. First, the effect of muscle length on developed force (F_dev_) and kinetics was assessed at four different muscle lengths (set as a percentage of optimal length) and a constant frequency of 0.5 Hz. After force and kinetics were measured at L_100_, the muscle length was reduced to 85% of L_100_ (L_85%_), then 90% (L_90%_), then 95% (L_95%_), and then back again to L_100_. Muscles were allowed to stabilize at each length before parameters were recorded. We assessed the effect of increasing stimulation frequencies (frequency-dependent activation) at L_100_ between 0.5 and 3 Hz, spanning the entire physiological range of a healthy adult human heart. Forces were allowed to reach a steady-state at each frequency (total of 6) before data were recorded (typically 2–3 minutes at each frequency). Lastly, the effects of β-adrenergic stimulation were assessed by a concentration-response curve with isoproterenol (1 nM– 1 μM) at a baseline stimulation frequency of 0.5 Hz and L_100_.

### Data analysis and statistics

A custom-made program in LabView (National Instruments) was used to analyze force and kinetic measurements. Muscle tensions were normalized to the cross-sectional area of the muscles and expressed as mN/mm^2^. Statistical comparison of the mean values of contractile forces and kinetic parameters between NF, FNH, and FH groups was performed via one-way analysis of variance (ANOVA), or by two‐way ANOVA for repeated measures. This comparison was then followed by Tukey’s *post hoc* multiple comparison test, comparing all pairs of columns. Unpaired two-tailed Student’s *t-*test was used to compare TSH levels between FNH and FH groups. Statistical significance was set at two‐tailed *P* < 0.05. All data are presented as means ± standard error of the mean (SEM).

## Results

### Baseline contraction and relaxation kinetics

**Fig 1:**
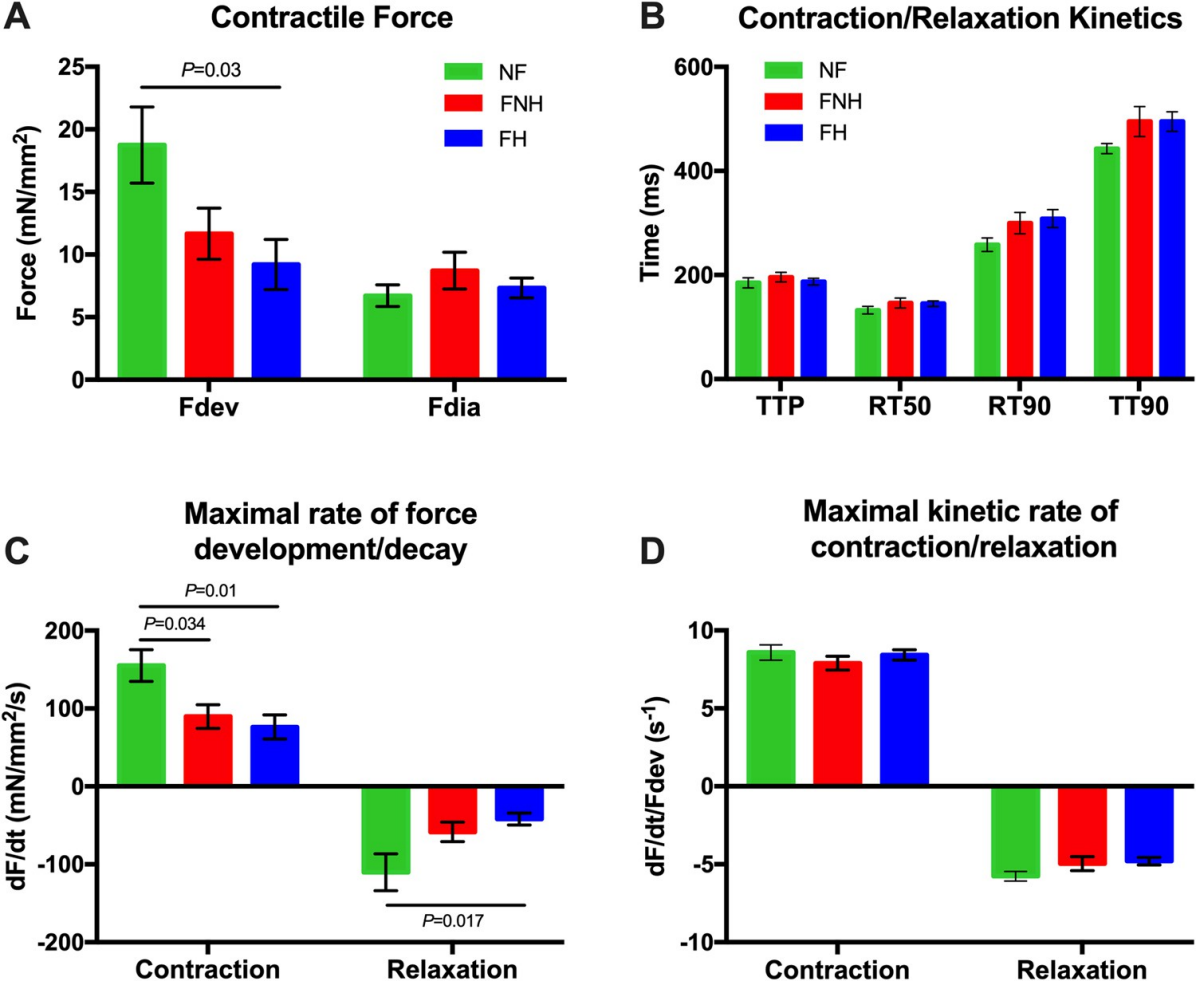
Contractile force and kinetics of NF trabeculae (*n* = 9) vs. failing trabeculae with no hypothyroidism (FNH; *n* = 9) and failing trabeculae with hypothyroidism (FH; *n* = 9) at baseline 1 Hz. **(A)** F_dev_ of NF trabeculae was significantly greater than that of FH trabeculae, while there was no significant difference in F_dia_ between the three groups. **(B)** TTP, RT50, RT90 and TT90 were not significantly different between groups. **(C) +**dF/dt was significantly prolonged in FNH and FH trabeculae compared to NF trabeculae, while -dF/dt was significantly slower in FH trabeculae compared to NF trabeculae. **(D) +**dF/dt/F_dev_ and -dF/dt/F_dev_ were not significantly different between all groups. Data are depicted as means ± SEM; One-way ANOVA followed by Tukey’s *post-hoc* test for multiple comparisons. +dF/dt, maximal rate of force development during contraction; -dF/dt, maximal rate of force decay during relaxation; +dF/dt/F_dev_, maximal kinetic rate of contraction; -dF/dt/F_dev_, maximal kinetic rate of relaxation; F_dev_, active developed force; F_dia_, diastolic force; FH, failing with hypothyroidism; FNH, failing with no hypothyroidism; NF, non-failing; RT50, time from peak tension to 50% relaxation; RT90, time from peak tension to 90% relaxation; TT90, time from stimulation to 90% relaxation time; TTP, time to peak tension.

Active developed force (F_dev_) of trabeculae isolated from FH hearts was the lowest among the groups and significantly different from NF trabeculae (9.2 ± 2.0 mN/mm^2^ vs. 18.7 ± 3.0 mN/mm^2^; *P* = 0.03), while there was no significant difference in diastolic force (F_dia_) between the three groups (Fig 1A). We analyzed the kinetic parameters of the same muscles, shown in Fig 1B, where we detected no significant difference in time from electrical stimulation to peak tension (TTP) between NF, FNH, and FH trabeculae. Similarly, time from peak tension to 50% relaxation (RT50) and time from peak tension to 90% relaxation (RT90) were also non-significant between the three groups. Total twitch time, calculated as the time from stimulation to 90% relaxation time (note, 100% relaxation time cannot be accurately assessed as force asymptotically approaches resting tension), was also not significant. We found that trabeculae isolated from FNH hearts (90 ± 15 mN/mm^2^/s; *P* = 0.034) and FH (76 ± 16 mN/mm^2^/s; *P* = 0.01) had a significantly slower rate of tension rise (**+**dF/dt) compared to that of NF trabeculae (155 ± 20 mN/mm^2^/s). Rate of tension decline (-dF/dt) was significantly prolonged in FH hearts (-42 ± 8 mN/mm^2^/s; *P* = 0.017) compared to NF (-110 ± 24 mN/mm^2^/s), while there was no significant difference between the two failing groups (Fig 1C). However, the derivative of force (dF/dt) is highly dependent on the level of force. Both **+**dF/dt and -dF/dt were then normalized to the developed twitch tensions to obtain pure kinetic parameters [36]. The maximal kinetic rate of contraction (**+**dF/dt/F_dev_) and the relaxation parameter (-dF/dt/F_dev_) were not significantly different between groups (Fig 1D).

### Impact of muscle length on contraction/relaxation and kinetics

**Fig 2:**
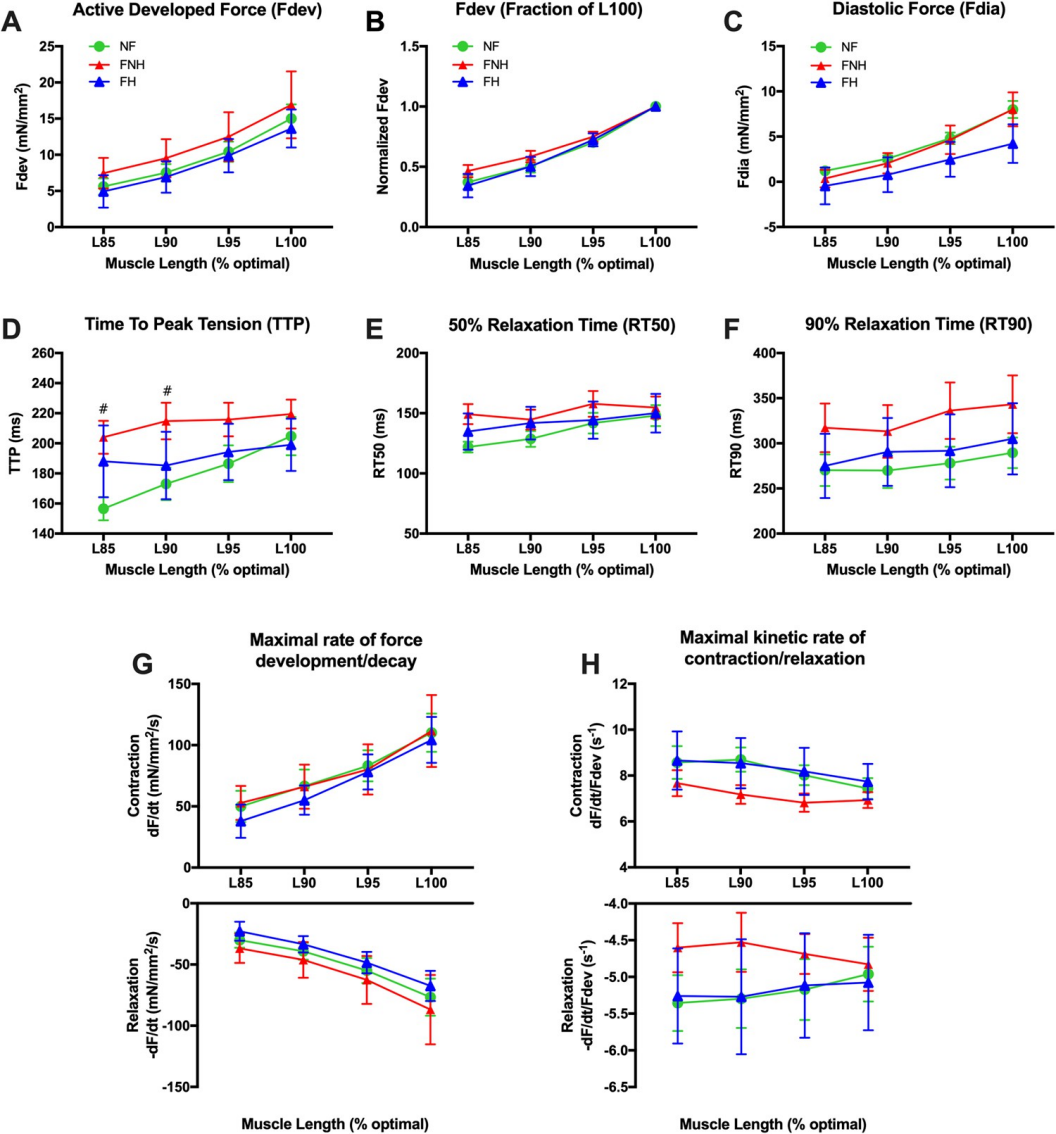
Length-dependent activation in NF trabeculae (*n* = 9) vs. failing trabeculae with no hypothyroidism (FNH; *n* = 9) and failing trabeculae with hypothyroidism (FH; *n* = 4). **(A)** Length dependent force development was virtually identical in all groups. **(B)** Same data as panel A, plotted to each muscle’s individual maximum to allow equal weight of each muscle in the statistical analysis. **(C)** F_dia_ increased by increasing the muscle length, and there was no difference in the degree of change in force between the three groups in response to length increase. **(D)** TTP in NF myocardium was markedly slowed down as muscle length increased rather than the two failing groups, while trabeculae isolated from FNH hearts showed a prolonged TTP overall different muscle lengths compered to both NF and FH hearts and became significantly different compared to NF at L_85_ (*P* = 0.013) and L_90_ (*P* = 0.032). **(E)** RT50 decreased as muscles were shortened from L_100_ to L_85_ and the degree of shortening was much obvious in NF group. **(F)** RT90 was not significantly different between the three groups at each respective muscle length and was faster as muscles were shortened. **(G)** +dF/dt and -dF/dt were prolonged in NF and failing groups as muscles were shortened and were not significantly different between groups. **(H) +**dF/dt/F_dev_ was sped up as muscles were shortened with no difference between groups. With the exception of trabeculae isolated from FNH hearts, -dF/dt/F_dev_ was also sped up in both NF and FH groups. Data are depicted as means ± SEM; A two-way repeated measures ANOVA followed by Tukey’s *post hoc* test for multiple comparisons; * indicates *P* < 0.05 significant difference between NF and FH groups; ^#^ indicates *P* < 0.05 significant difference between NF and FNH groups at corresponding muscle length. L100, optimal muscle length; L85, 85% of L100; L90, 90% of L100; L95, 95% of L100; other abbreviations as in Fig 1.

In agreement with previous studies [37,38], increasing muscle length from L_85_ to L_100_ stepwise increased developed tensions in response to a change in muscle length in all groups to the same degree (at a constant frequency of 0.5 Hz) (Fig 2A). To investigate the true length-dependency, we plotted the data in Fig 2B, where developed forces were normalized to the maximal force of the muscle at L_100_. This normalization helps eliminate the effect of the absolute forces of the stronger muscles, which strongly control this presentation mode. Our results showed the linearity of this relationship with no difference between the three groups. Moreover, there was no difference in the degree of change in F_dia_ between groups in response to length increase (Fig 2C). Trabeculae from FNH hearts had a longer TTP overall different muscle lengths than those of NF and FH hearts, and this difference was statistically significant at L_85_ and L_90_ (204 ± 11 ms and 215 ± 12 ms, respectively) vs. NF trabeculae (157 ± 8 ms; *P* = 0.013 and 173 ± 11 ms; *P* = 0.032, respectively) (Fig 2D). RT50 and RT90 were not significantly different between groups at each respective muscle length (Fig 2E and 2F). Furthermore, **+**dF/dt and -dF/dt were faster as muscle length increased with no significant difference between NF and both failing groups at each respective muscle length (Fig 2G). In all groups, **+**dF/dt/F_dev_ was slowed down as muscle length increased. Similarly, -dF/dt/F_dev_ was slowed down except in FNH group where it sped up (Fig 2H).

### Impact of frequency on contraction/relaxation and kinetics

**Fig 3:**
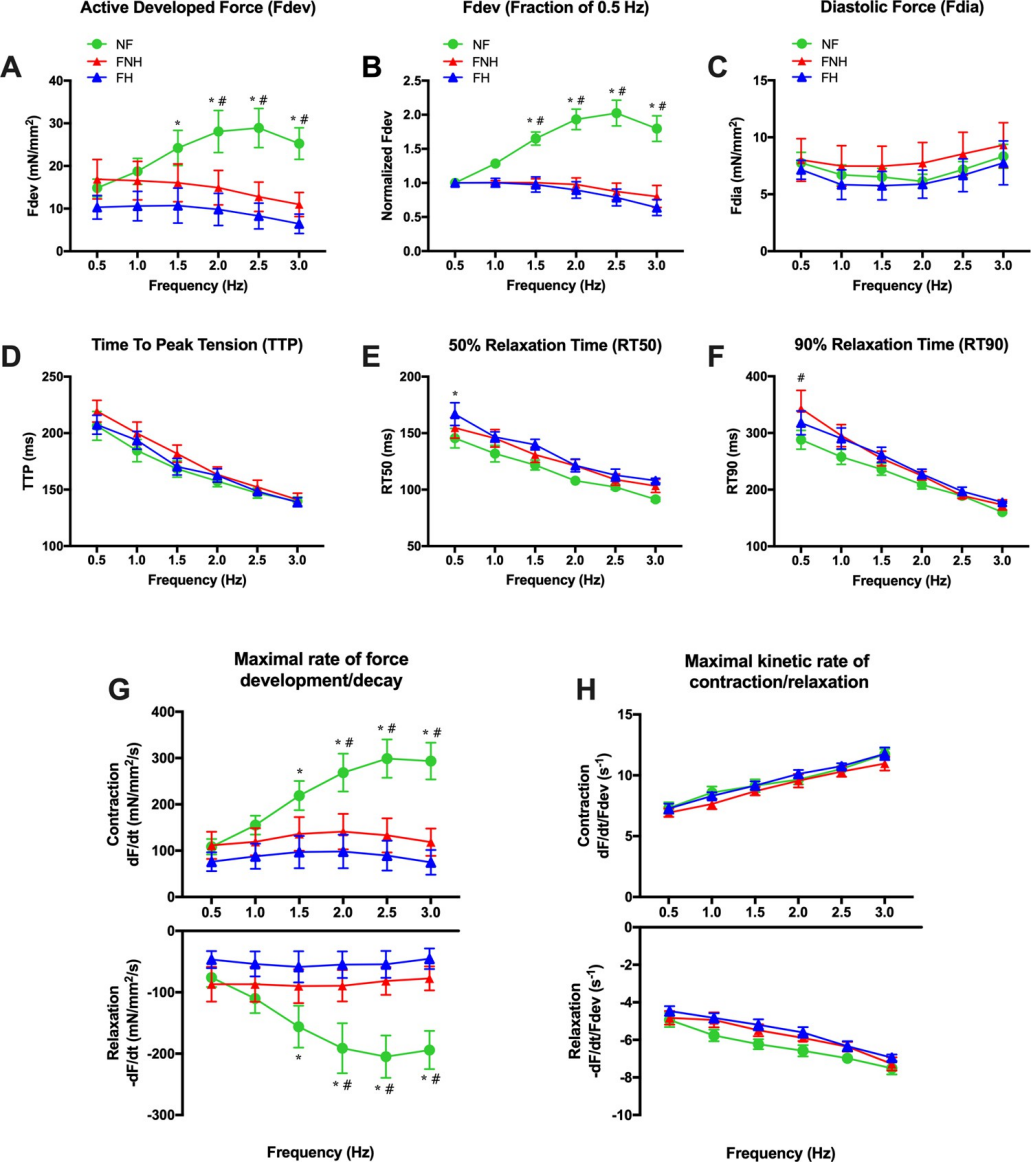
Frequency-dependent activation in NF trabeculae (*n* = 9) vs. failing trabeculae with no hypothyroidism (FNH; *n* = 9) and failing trabeculae with hypothyroidism (FH; *n* = 4). **(A)** Frequency-dependent regulation of force was significantly different between trabeculae isolated from NF, FNH and FH hearts, where it was significantly positive in NF myocardium. **(B)** Same data as panel A, plotted to each muscle’s force at 0.5 Hz to allow equal weight of each muscle in the statistical analysis. At all frequencies, force in NF trabeculae exceeded the force at the 0.5 Hz baseline. **(C)** F_dia_ slightly declined at 1 and 2 Hz in NF and failing groups. In all groups, F_dia_ was higher at 3 Hz than that of 0.5 Hz. **(D)** TTP shortened upon increasing frequency of stimulation in all groups with no differences between NF and both failing groups at each respective frequency. **(E)** RT50 decreased as frequency increased from 0.5 Hz to 3 Hz in all three groups. **(F)** RT90 was not significantly different between the three groups at each respective frequency of stimulation and was shorter at higher frequency (3 Hz). **(G)** +dF/dt and -dF/dt were sped up in NF and failing groups as frequency increased and were significantly faster in NF group compared to both failing groups at 1.5–3 Hz. **(H) +**dF/dt/F_dev_ and -dF/dt/F_dev_ were faster in all groups over the entire frequency range. However, the acceleration of this kinetic rate was equal in all three groups. Data are depicted as means ± SEM; A two-way repeated measures ANOVA followed by Tukey’s *post hoc* test for multiple comparisons; * indicates *P* < 0.05 significant difference between NF and FH groups; ^#^ indicates *P* < 0.05 significant difference between NF and FNH groups at corresponding stimulation frequency. FFR, force-frequency relationship; other abbreviations as in Fig 1.

Our data showed that NF myocardium typically displayed a positive force-frequency relationship (FFR) where F_dev_ increased between 0.5 and 2.5 Hz, while both FNH and FH myocardium had a flat or negative FFR between 0.5 and 2 Hz (Fig 3A). F_dev_ of both failing groups was significantly lower compared to NF group at frequencies ranging from 1.5–3 Hz. We normalized developed forces to 0.5 Hz to assess the changes in developed forces at higher frequencies and determine FFR (Fig 3B). This analysis also showed a positive FFR in NF myocardium and flat FFR between 0.5–2 Hz in both failing groups. The diastolic force was slightly decreased at 1 and 2 Hz in NF and failing groups, while it was much higher at 3 Hz than at 0.5 Hz (Fig 3C). No major alterations in most of the kinetic parameters were observed between groups. TTP shortened upon the increasing frequency of stimulation in all three groups. There were no differences between NF and the two failing groups at each respective frequency of stimulation (Fig 3D). In NF and failing groups, RT50 and RT90 shortened across the tested frequencies (Fig 3E and 3F). Both **+**dF/dt and -dF/dt of NF, as well as failing groups, sped up with frequency increase in all groups and were statistically significant between NF group and the two failing groups at 1.5–3 Hz (*P* < 0.05) (Fig 3G). Furthermore, both **+**dF/dt/F_dev_ and -dF/dt/F_dev_ were significantly faster in all groups upon increase the stimulation frequency (Fig 3H).

### Impact β-adrenergic stimulation on contraction/relaxation and kinetics

**Fig 4:**
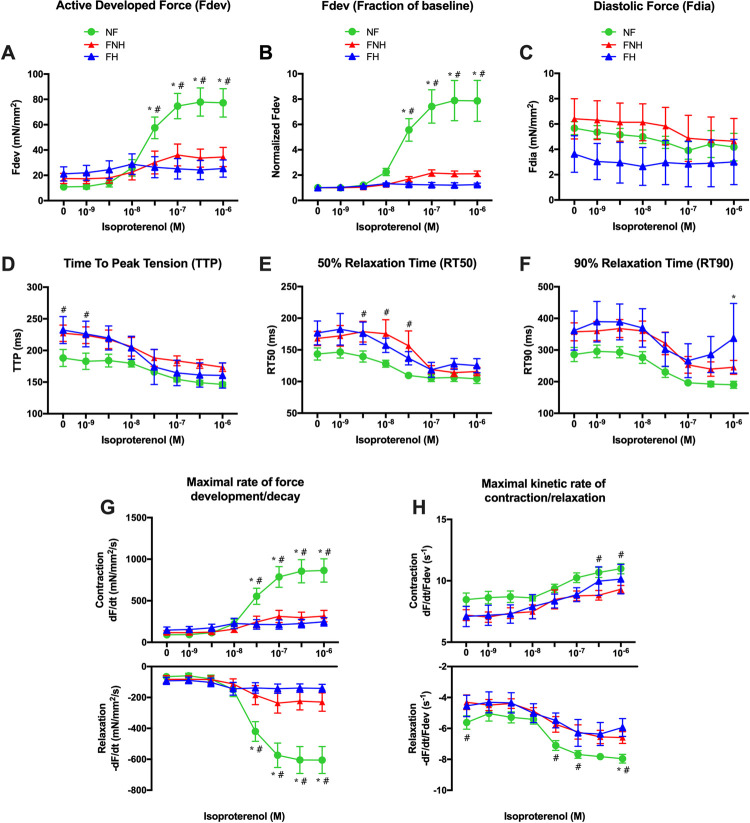
β-adrenergic stimulation in NF trabeculae (*n* = 9) vs. failing trabeculae with no hypothyroidism (FNH; *n* = 9) and failing trabeculae with hypothyroidism (FH; *n* = 4). **(A)** F_dev_ was increased in NF trabeculae upon addition of isoproterenol in a concentration dependent manner and was significantly greater than the increase in both failing groups. **(B)** Same data as panel A, plotted to each muscle’s force at baseline to allow equal weight of each muscle in the statistical analysis. **(C)** F_dia_ slightly decreased in NF and both failing groups over the concentration range with no significant difference between the three groups. **(D)** TTP shortened almost similarly in all three groups upon increasing isoproterenol concentration. **(E)** RT50 slightly decreased as concentration increased from 1 nM to 1 μM in all three groups. **(F)** RT90 was not significantly different between the three groups over the concentration range. **(G)** +dF/dt and -dF/dt were sped up in NF and failing groups as isoproterenol concentration increased and were significantly faster in NF group compared to both failing groups at the concentration range of 30 nM—1 μM. **(H)** NF myocardium showed faster +dF/dt/F_dev_ and -dF/dt/F_dev_ throughout the concentration-response protocol compared to both failing groups with significantly faster +dF/dt/F_dev_ at 0.3 μM and 1 μM and significantly faster -dF/dt/F_dev_ at 30 nM, 0.1 μM and 1 μM vs. FNH myocardium. Data are depicted as means ± SEM; A two-way repeated measures ANOVA followed by Tukey’s *post hoc* test for multiple comparisons; * indicates *P* < 0.05 significant difference between NF and FH groups; ^#^ indicates *P* < 0.05 significant difference between NF and FNH groups at corresponding isoproterenol concentration. Abbreviations as in Fig 1.

NF myocardium exhibited significantly increased responses than both failing groups under full β-adrenergic stimulation (Fig 4A). Relative to baseline (no isoproterenol), isoproterenol-induced force development was similarly depressed in both failing groups, while statistical significance was reached at 30 nM, 0.1 μM, 0.3 μM, and 1 μM compared to NF myocardium (Fig 4B). Diastolic tension slightly decreased in the NF group and both failing groups over the concentration range, as shown in Fig 4C, without any significant difference between groups. Kinetics accelerated similarly in each group (curve shape is the same/highly similar), as illustrated for TTP (Fig 4D), RT50 (Fig 4E), and RT90 (Fig 4F) were shortened down almost similarly in all the three groups. In NF trabeculae, both **+**dF/dt and -dF/dt were sped up over the concentration range (Fig 4G). In failing groups, statistical significance compared to NF group was reached at 30 nM, 0.1 μM, 0.3 μM, and 1 μM. **+**dF/dt/F_dev_ was much faster throughout the concentration-response protocol in NF trabeculae compared to both failing groups and showed a significant difference compared to FNH at 0.3 μM and 1 μM. -dF/dt/F_dev_ was significantly faster in NF group vs. FNH group, mostly at higher concentrations (Fig 4H).

### Clinical information

Clinical information was available for all HF patients with and without hypothyroidism in our study. The mean age was 56 years, with 22% female. In a recent study published by our laboratory, a total of 112 hearts [non-failing (*n* = 58) and failing (*n* = 54)] were used in an assessment of LV isolated muscle function. The results of unpaired two-tailed *t*-test (not reported) revealed that gender did not impact force within NF or even failing myocardium. Moreover, the interaction between the effects of gender and pathology was not significant on the Fdev parameter [33]. Accordingly, limited representation of female subjects in this cohort has no effect on the contractile force outcomes.

The New York Heart Association classification was predominantly class IV (88%) with a minority of class III (11%). All patients with hypothyroidism received levothyroxine treatment with a dose range of 25–200 μg/day. Likely due to this therapy, HF with hypothyroidism had TSH levels ranging from 0.05–8.19 mU/L, which were not significantly different from TSH levels of HF patients who had no thyroid dysfunction (4.28 ± 1.22 mU/L vs. 3.12 ± 0.49 mU/L; *P* = 0.41). Information on whether patients started levothyroxine treatment before or after diagnosis with HF was not available.

## Discussion

We reported here for the first time that hypothyroidism has no additional impact on the force of contraction and kinetics of contraction/relaxation of LV intact human myocardial muscle preparation isolated from end-stage failing heart different than the recognized alterations usually detected in such parameters in any end-stage failing heart without underlying thyroid dysfunction.

Our study was performed on end-stage HF, which is the most severe class of the four classes of HF as described in The New York Heart Association. Absence of any difference between failing hearts with or without hypothyroidism, may possibly be due to the profound effects of the advanced stage of HF that concealed any changes between the groups. Still, we cannot rule out the possibility of differences that may have been present at earlier stages of HF.

The relationship between hypothyroidism and heart diseases is not unidirectional. HF can lead to the down-regulation of the thyroid hormone signaling system in the heart [15]. In the failing heart, decreases of nuclear TR levels occur. In addition, HF may also alter the metabolism of thyroid hormones. Numerous reports from clinical and animal studies have documented altered thyroid hormone metabolism with low serum levels of T3 and T4 with HF in the context of the non-thyroidal illness syndrome. The hypoxia and the inflammatory response to HF are able to reduce the type 2 deiodinase (D2) activity in the cardiomyocytes, which results in a decrease of intracellular T3 bioavailability [39]. In a rodent model of cardiac disease, overexpression of D2 in the heart was able to enhance cardiac contractility while preventing cardiac functional deterioration and altered gene expression after pressure overload [40]. Hypoxia also can induce the expression of D3 deiodinase, which converts T4 to the deactivated rT3 metabolite in cardiomyocytes. In another study using rat model of right ventricular (RV) hypertrophy and failure, D3 activity increased in the chronically overloaded RV, whereas no changes were observed in the LV of the same heart [41]. The abnormalities in TRs and the decreased deiodinase activities could both lead to the development of important changes in cardiac gene expression, which are like those observed in hypothyroidism. Based on current findings, the American Heart Association in 2013 issued guidelines recommending that doctors test thyroid hormone levels in all patients with HF [42].

Clinical information for FH patients in our study revealed that they were all receiving THs replacement therapy, levothyroxine, but we have limited information about the circumstances of their diagnoses with HF and hypothyroidism as well as when they started receiving levothyroxine treatment. Our statistical analysis did not show significant differences in FH patients’ TSH levels compared to the FNH patients, which possibly reflects the efficiency and accuracy of the thyroid hormone doses used in treating these patients. Previous studies have reported that TH replacement therapy in patients with hypothyroidism generally may lower peripheral vascular resistance, reverse atherosclerosis, and improve myocardial perfusion [43]. Treatment of overt hypothyroidism and subclinical hypothyroidism using levothyroxine has proven its ability to prevent progressive LV dysfunction by improving systolic and diastolic dysfunctions, systemic vascular resistance, and endothelial function, which can improve cardiac output (CO) thereby increasing CO and stroke volume [44,45]. Moreover, levothyroxine in replacement doses is recommended by international guidelines and expert opinions in patients with serum TSH above 10 mU/L [46]. Since patients in our study underwent screening for their heart conditions, they were all receiving treatment for their thyroid problems. It was unfeasible to find samples from HF patients who got diagnosed with hypothyroidism and didn’t receive any treatment for that problem. Lacking such data, didn’t allow us to confirm the possible role of levothyroxine in controlling the anticipated hypothyroidism myocardial alterations in FH group. Indeed, more studies are still needed to investigate the effects of THs supplementation such as levothyroxine on contractile force and kinetic parameters of failing human myocardium and explore its full potential in improving cardiovascular performance and cardiovascular outcomes of HF associated with hypothyroidism.

Our results showed that the active developed force of contraction at resting heart rate (1 Hz and L_100_) was significantly lower in FH compared to NF group at resting. The level of developed force in failing preparations compared to non-failing can be decreased as shown in a previous study performed on muscle strip and myocytes [47], increased as in isolated myocytes preparation [48] or reported no significant difference as we shown before in trabeculae [49]. Changes in myofilament calcium sensitivity are suggested to be one of the reasons behind altered force development capability. Since we did not measure this parameter, we cannot assume that the difference found in our study directly relates to hypothyroidism. Our present data also showed that there were no differences in the pure kinetic parameters (**+**dF/dt/F_dev_ and -dF/dt/F_dev_) between the NF and both failing groups when measured at 1 Hz and L_100_. Previous studies on LV muscles of failing human myocardium confirm these findings [50,51]. Recent studies from our laboratory showed that when assessed in high numbers, the statistical power may be sufficient to detect small differences in contractile kinetics [32,49], but given the low numbers of hypothyroid hearts available, it was not feasible to obtain larger number of hearts where subtle differences may become statistically significant.

Length-dependent regulation is one of the main regulatory pathways that impacts cardiac force. In our study, the length-dependent activation mechanism in both failing groups was completely preserved, both in the regulation of active force development and in the regulation of kinetics. With an increase in muscle length, all three groups responded to a similar quantitative and qualitative increase in force and slowing of kinetics similar to what had been reported before in different studies [32,52].

The LV myocardium of failing hearts (with or without thyroid dysfunction) in the present study had the expected blunted FFR, a classical hallmark that has been extensively observed in failing human myocardium [32,48,49]. Alterations in calcium handling mechanisms are mainly responsible for such abnormal FFR [53]. Rates of contraction and relaxation were significantly slower in failing myocardium over the entire range of stimulating frequencies. Defective calcium homeostasis in failing myocardium is partially responsible for slow relaxation and poor contractile response to increasing stimulation frequency.

Finally, in close agreement with previous studies [32,49], the β-adrenergic response was also blunted in both failing groups. The mechanisms responsible for β-adrenergic desensitization have been attributed to receptor desensitization and/or downregulation following prolonged exposure to catecholamines [54]. A significant slow-down of contraction and relaxation rates was observed upon stimulation, similar to our results in frequency-dependent activation as previously reported [32].

### Limitations of the study

In order to detect small differences in contractile responses or changes in kinetics, in combination with the large variability (mostly due to genetic variety) in patient samples, much larger *n*-numbers of samples would be needed. However, given the fact that this study took over 8 years to complete, a study with the same statistical power that we completed in a decade-long study recently reported [32], it would take several decades to complete. Hence, we cannot exclude subtle differences in force or kinetics at the end-stage of HF between patients with hypothyroidism, and those without.

It was unfeasible to find samples from HF patients who got diagnosed with hypothyroidism and didn’t receive any treatment to compare against and confirm if levothyroxine played a role in controlling the anticipated hypothyroidism myocardial alterations in FH group in our study.

We did not assess protein levels and phosphorylation levels in this study. The phosphorylation status of several contractile proteins has been shown previously to be impacted by both pre-load [55], and by frequency [56] and this phosphorylation profile is impacted in disease [57]. Per se, a correlative study on the impact of phosphorylation on force development and regulation needs samples at different contractile states (different lengths, frequencies, and beta-stimulation), which was not possible in our studies. In addition, the phosphorylation status of procured quiescent tissue, under unloaded conditions, may thus provide, at best, a weak substitute for actual experiments on tissue in contractile homeostasis. Although we have collected several muscles at homeostasis, we can unfortunately only freeze each muscle in this study under 1 condition at the very end of the study (typically in presence of maximal β-adrenergic stimulation). Studies with human tissue frozen at different homeostasis (i.e., at different pre-load, and at different frequencies), are needed in the future to shed more light on the underlying mechanisms”.

### Conclusions

Hypothyroidism didn’t show any additional impact on the force of contraction and kinetics of contraction/relaxation in end-stage failing hearts. Absence of any difference may possibly be due to the profound effects of the advanced stage of HF that concealed any changes between the two groups. Still, we cannot exclude the possibility of differences that may have been present at earlier stages of HF. Therefore, future studies are critically needed to investigate the impact of hypothyroidism on the earlier stages. Since it was unfeasible to find samples from HF patients who got diagnosed with hypothyroidism and not receiving treatment to compare against, we couldn’t confirm the possible role of levothyroxine in controlling the anticipated hypothyroidism myocardial alterations in the FH group in our study. The effects of THs supplementation such as levothyroxine on contractile force and kinetic parameters of such failing myocardium, still needs further exploration.

## Supporting information

S1 Table TSH level of heart failure patients with no thyroid dysfunction/with hypothyroidism and their Levothyroxine treatment doses. (DOCX)

S1 Dataset (XLSX)

S2 Dataset (XLSX)

S3 Dataset (XLSX)
