# Supplementary material for: Effect of hypothyroidism on contractile performance of isolated end-stage failing human myocardium
Source: PLoS One. 2022 Apr 11;17(4):e0265731. doi: 10.1371/journal.pone.0265731 (PMC9000031; doi:10.1371/journal.pone.0265731)
Supplement: S1 Table — (DOCX) [file pone.0265731.s001.docx]

**S1 Table. TSH level of heart failure patients with no thyroid dysfunction/with hypothyroidism and their Levothyroxine treatment doses.**

| ID # | Failing  Category | Sex | TSH level mU/L | | | Levothyroxine dose µg/day |
| --- | --- | --- | --- | --- | --- | --- |
| Failing human hearts with no hypothyroidism (FNH; *n* = 9) | | | | | | |
| FNH1 | ICM | M | 5.242 | | - | |
| FNH2 | NICM | F | - | | - | |
| FNH3 | NICM | M | 2.65 | | - | |
| fNH4 | NICM | M | 3.5 | | - | |
| fNH5 | NICM | M | 0.735 | | - | |
| fNH6 | NICM | M | 4.34 | | - | |
| fNH7 | NICM | F | 2.89 | | - | |
| fNH8 | NICM | F | 3.515 | | - | |
| fNH9 | NICM | M | 2.105 | | - | |
| Failing human hearts with hypothyroidism (FH; *n* = 9) | | | | | | |
| FH1 | ICM | M | 5.95 |  | | 25 |
| Fh2 | ICM | M | 4.16 |  | | 200 |
| FH3 | ICM | M | 1.995 |  | | 50 |
| FH4 | ICM | F | 10.366 |  | | 25 |
| FH5 | ICM | M | 0.217 |  | | 150 |
| FH6 | NICM | M | 8.19 |  | | 100 |
| FH7 | NICM | M | 6.245 |  | | 88 |
| FH8 | NICM | M | 0.9 |  | | 50 |
| FH9 | NICM | F | 0.048 |  | | 156 |
